# Integrative Discovery of Epigenetically Derepressed Cancer Testis Antigens in NSCLC

**DOI:** 10.1371/journal.pone.0008189

**Published:** 2009-12-04

**Authors:** Chad A. Glazer, Ian M. Smith, Michael F. Ochs, Shahnaz Begum, William Westra, Steven S. Chang, Wenyue Sun, Sheetal Bhan, Zubair Khan, Steven Ahrendt, Joseph A. Califano

**Affiliations:** 1 Department of Otolaryngology—Head and Neck Surgery, Johns Hopkins Medical Institutions, Baltimore, Maryland, United States of America; 2 Division of Oncology Biostatistics, Department of Oncology, Johns Hopkins Medical Institutions, Baltimore, Maryland, United States of America; 3 Department of Pathology, Johns Hopkins Medical Institutions, Baltimore, Maryland, United States of America; 4 Department of Surgery, University of Pittsburgh Medical Center, Pittsburgh, Pennsylvania, United States of America; 5 Milton J Dance Head and Neck Center, Greater Baltimore Medical Center, Baltimore, Maryland, United States of America; University of Barcelona, Spain

## Abstract

**Background:**

Cancer/testis antigens (CTAs) were first discovered as immunogenic targets normally expressed in germline cells, but differentially expressed in a variety of human cancers. In this study, we used an integrative epigenetic screening approach to identify coordinately expressed genes in human non-small cell lung cancer (NSCLC) whose transcription is driven by promoter demethylation.

**Methodology/Principal Findings:**

Our screening approach found 290 significant genes from the over 47,000 transcripts incorporated in the Affymetrix Human Genome U133 Plus 2.0 expression array. Of the top 55 candidates, 10 showed both differential overexpression and promoter region hypomethylation in NSCLC. Surprisingly, 6 of the 10 genes discovered by this approach were CTAs. Using a separate cohort of primary tumor and normal tissue, we validated NSCLC promoter hypomethylation and increased expression by quantitative RT-PCR for all 10 genes. We noted significant, coordinated coexpression of multiple target genes, as well as coordinated promoter demethylation, in a large set of individual tumors that was associated with the SCC subtype of NSCLC. In addition, we identified 2 novel target genes that exhibited growth-promoting effects in multiple cell lines.

**Conclusions/Significance:**

Coordinated promoter demethylation in NSCLC is associated with aberrant expression of CTAs and potential, novel candidate protooncogenes that can be identified using integrative discovery techniques. These findings have significant implications for discovery of novel CTAs and CT antigen directed immunotherapy.

## Introduction

It is well known that CTAs are overexpressed in various tumor types, with little or no expression in normal human tissue; however, the mechanism of this differential expression is not well understood [1]. Epigenetic changes including alterations in promoter methylation have been associated with cancer-specific expression differences in human malignancies, including non-small cell lung carcinoma (NSCLC) [2], [3], [4], [5], [6], [7]. Promoter hypermethylation has primarily been considered as a mechanism of tumor suppressor gene inactivation; however, global genomic hypomethylation has been reported in almost all tumors [2], [4], [8], [9]. It is also known that CTAs, especially those encoded by the X chromosome (CT-X antigens), are expressed in association with promoter demethylation or whole genomic hypomethylation [10], [11], [12].

Lung cancer is the leading cause of cancer-related deaths worldwide, with over 213,000 new cases and 160,000 deaths reported in 2007; NSCLC accounts for approximately 75% of these cases [13]. The high mortality rate in NSCLC is attributable to diagnosis at an advanced stage, a high rate of recurrence despite definitive locoregional management, and the fact that no therapies for recurrent lung cancer have been associated with improved long-term survival [6].

One approach to improve NSCLC mortality has been the development of cancer vaccines which aim to induce a host immune response against tumor cells. CTAs are attractive targets for tumor immunotherapy because of their restricted expression patterns in normal human tissue. For example, NY-ESO-1 and MAGEA3 are currently undergoing clinical trials in various human malignancies, including NSCLC. Investigators have suggested that combined use of coordinately expressed CTAs and other associated antigens could aid in the design of more effective, polyvalent immunotherapeutic protocols in NSCLC and other tumor types; however, there is currently very little known about the coexpression patterns of these genes [14], [15], [16].

To date, a comprehensive, genome-wide approach to identify coordinately expressed CTAs and other differentially expressed genes activated by promoter demethylation in NSCLC has not been conducted. In this study, we used a novel, integrative epigenetic screening approach combining 5-aza-deoxycytidine and trichostatin A (5-aza/TSA) treatment of normal lung cells and mRNA microarray expression analysis of primary tissue to identify genes that are both activated by DNA hypomethylation and differentially upregulated in a coordinated fashion in NSCLC. We were able to define a set of coordinately expressed CTAs and novel, growth promoting genes that are activated in association with coordinated promoter demethylation. These data have implications for the mechanism of activation of CTAs, design of immunotherapeutic strategies, as well as identification of potential protooncogenes and novel biologic targets for gene directed therapies for NSCLC.

## Materials and Methods

### Histopathology

All samples were analyzed by the pathology department at Johns Hopkins Hospital. Tissues were obtained via Johns Hopkins Institutional Review Board approved protocol NA_00001911. Written informed consent was obtained from each subject prior to the use of their tissue for scientific research. Tumor and normal lung tissues from surgical specimens were frozen in liquid nitrogen immediately after surgical resection and stored at −80°C until use. Normal samples were microdissected and DNA prepared from normal lung parenchyma. Tumor samples were confirmed to be NSCLC and subsequently microdissected to yield at least 80% tumor cells. Tissue DNA and RNA was extracted as described below.

### 5Aza-dC and TSA Treatment of Cells

We treated normal human lung cell lines (NHBE and SAEC, Lonza, Walkersville, MD) in triplicate with 5-aza-deoxycytidine (a cytosine analog which cannot be methylated) and trichostatin A (a histone deacetylase inhibitor) as described previously [17]. Briefly, cells were split to low density (2.5×10^5^ cells and 6×10^5^/100 mm dish for SAEC and NHBE respectively) 24 hours before treatment. Stock solutions of 5Aza-dC (Sigma, St. Louis, MO) and TSA (Sigma) were dissolved in 50% acetic acid and 100% ethanol, respectively. Cells were treated with 5 uM 5Aza-deoxycytidine for 72 hours and 300 nmol/L TSA for the last 24 hours. Baseline expression was established by mock-treated cells with the same volume of acetic acid or ethanol in triplicate.

### RNA Extraction and Oligonucleotide Microarray Analysis

Total cellular RNA was isolated using Trizol (Life Technologies, Gaithersburg, MD) and the RNeasy kit (Qiagen, Valencia, CA) according to the manufacturer's instructions. We carried out oligonucleotide microarray analysis using the GeneChip U133 Plus 2.0 Affymetrix expression microarray (Affymetrix, Santa Clara, CA). Samples were converted to labeled, fragmented, cRNA per the Affymetrix protocol for use on the expression microarray. Signal intensity and statistical significance was established for each transcript using dChip version 2005 to initially analyze and normalize the array data and then Significance Analysis of Microarrays (SAM) [18]. SAM output was calculated at a d-value of 1.126 yielding a false discovery rate and d-score cutoff of 5.065% and 1.885. This identified a total of 12,132 upregulated candidate genes after 5Aza-dC/TSA treatment. All microarray data is MIAME compliant, and the raw data has been deposited in a MIAME compliant database, GEO, as detailed by the MGED Society.

### Public Datasets

The public databases used in this study were the University of California Santa Cruz (UCSC) Human Genome reference sequence and the annotation database from the March 2006 freeze (hg18). We obtained 40 normal lung and 111 NSCLC expression microarrays from the expO datasets (all performed on the Affymetrix U133 Plus 2.0 mRNA expression platform) available online as part of the Gene Expression Omnibus (GEO/NCBI). Accession numbers for these arrays are GSE1643 and GSE3141 respectively. The microarrays from normal tissue and tumor were first normalized for COPA analysis using dChip version 2005.

### Cancer Outlier Profile Analysis (COPA)

We applied COPA to our cohort of 151 tissues (111 tumors, 40 normals), with each gene expression data set containing 54,613 probe sets from the Affymetrix U133 Plus 2.0 mRNA expression platform. Briefly, gene expression values were median centered, setting each gene's median expression value to zero. The median absolute deviation (MAD) was calculated and scaled to 1 by dividing each gene expression value by its MAD. Of note, median and MAD were used for transformation as opposed to mean and standard deviation so that outlier expression values do not unduly influence the distribution estimates, and are thus preserved post-normalization. Finally, the 75th, 90th, and 95th percentiles of the transformed expression values are calculated for each gene and then genes were rank-ordered by their percentile scores, providing a prioritized list of outlier profiles. For the purposes of our rank-list, the 90th percentile for tumors was chosen based on sample-size analysis (111 tumors, 40 normals). Normal tissue that had a 95th percentile >2 was eliminated from our rank list. A total of 35,764 transcripts met the above criteria and were ranked. For details of the method refer to Tomlins et. al [19].

### Integrative Epigenetics

We ranked target genes from the Affymetrix U133 Plus 2.0 mRNA expression platform by COPA upregulation at the 90^th^ percentile (from 111 tumors and 40 normal tissues). The U133 Plus 2.0 mRNA expression platform (Affymetrix, Santa Clara California) has approximately 55,000 probe sets. A second rank list was produced by ranking genes in descending order of their d-score as computed by SAM following 5-aza/TSA treatment of normal lung cell lines (NHBE and SAEC). A third rank list was computed using 111 NSCLC and an additional expO dataset with 79 additional NSCLC primary tumor tissues also run on the Affymetrix Human Genome U133 Plus 2.0 mRNA expression platform. In these 190 primary NSCLC samples, we correlated BORIS expression patterns within each tumor with expression of all transcripts incorporated in the U133 Plus 2.0 array by calculating a correlation coefficient using Excel. All genes were then ranked based on the strength of the correlation between their expression and that of BORIS expression across all 190 samples. These three sources of information (gene set demonstrating upregulation with 5-aza, COPA score, and BORIS correlation) were combined by using a rank product (x*y*z). These three rankings were combined to rank all targets and permutation of the data was used to establish significance with a threshold of α = 0.005, yielding 290 significant genes. Genomic sequences were obtained for 122 of these genes using the UCSC genome browser, and the presence of CpG islands in the promoters or first intron of these genes was determined by MethPrimer which relies on GC content of >50%, >100 bp, >0.6 observed to expected CG's [20].

### DNA Extraction

Samples were centrifuged and digested in a solution of detergent (sodium dodecylsulfate) and proteinase K, for removal of proteins bound to the DNA. DNA was purified by phenol-chloroform extraction and ethanol precipitation. The DNA was subsequently resuspended in 500 µL of LoTE (EDTA 2.5 mmol/L and Tris-HCl 10 mmol/L) and stored at −80°C until use.

### Bisulfite Treatment and Sequencing

2 ug of DNA from 28 NSCLCs and 11 normal lung tissues were subjected to bisulfite treatment using the EpiTect® Bisulfite Kit (Qiagen, Valencia, CA) according to the manufacturer's instructions. This bisulfite-modified DNA was then stored at −80°C. Subsequently, bisulfite-treated DNA was amplified using primers designed by MethPrimer to span areas of CpG islands in the promoter or first intron [20]. Primer sequences were designed to not contain CG dinucleotides. Primer sequences are available in Table S1. Touch down PCR was performed and products were gel-purified using the QIAquick Gel Extraction Kit (Qiagen, Valencia, CA), according to the manufacturer's instructions. Each amplified DNA sample was applied with nested primers to the Applied Biosystems 3700 DNA analyzer using BD terminator dye (Applied Biosystems, Foster City, CA).

### Quantitative RT-PCR

Total RNA extracted as described above and the concentration for each sample was measured. 1 ug of RNA was then used for cDNA synthesis performed using oligo-dt with the SuperScript First- Strand Synthesis kit (Invitrogen, Carlsbad, CA). The final cDNA products were used as the templates for subsequent RT-PCR with primers designed specifically for each candidate gene. 18s rRNA was examined to ensure accurate relative quantitation in quantitative RT-PCR. Each experiment was performed in triplicate using the TAqMan 7900 (ABI) real-time PCR machine and the QuantiFast SYBR Green PCR Kit (Qiagen, Valencia, CA) according to the manufacturer's instructions. Primer sequences are available in Table S1.

### Quantitative Unmethylation-Specific PCR (QUMSP)

To selectively amplify demethylated promoter regions in genes of interest, primers were designed using data from bisulfite sequencing of primary tumors which are complimentary only to bisulfite-converted sequences known to be demethylated in tumors. Primer combinations were validated using *in vitro* methylated and demethylated controls. These experiments were performed in triplicate using the TAqMan 7900 (ABI) real-time PCR machine with standard curves and normalization to Beta-Actin primers that do not contain CpG's in the sequence for quantitation. Primer sequences are available in Table S1.

### Transfection of Human Expression Vectors and AD Growth Assay

A full-length ORF cDNA of SBSN and NY-ESO-1 were obtained for transient transfections from Origene in a pCMV6-Entry vector (Rockville, Maryland). Cell lines were plated at 5×10^5^ cells/well using 6-well plates and transfected with either empty vector or vector containing the gene of interest using the FuGene 6 Transfection Reagent (Roche, Basel, Switzerland) according to the manufacturer's protocol. Cell Counting Kit-8 (CCK-8) (Dojindo; Rockville, Maryland) absorbance was measured by the Spectramax M2e 96-well fluorescence plate reader Molecular Devices (Sunnyvale, California). All AD growth experiments were performed in triplicate for all cell lines and vectors.

### Anchorage-Independent Growth Assay

Soft agar assays were conducted following transfection of cells with mammalian pCMV6-Entry expression vectors containing a G418 resistance cassette (Origene). Cells were counted and approximately 5000 were added into each 6 well plate. The bottom layer was composed of 0.5% agar, RPMI +10% FBS, while the cells were suspended in a top layer of 0.35% agar, RPMI +10% FBS and G418 (300 ug/ml). Soft agar assays were incubated at 37 degrees for 12 days. All experiments were performed in triplicate for all cell lines and vectors.

### Statistical Analysis

We looked for similarities in the methylation patterns between genes by performing an analysis of correlations between QUMSP readings on the genes across all samples. Spearman's correlation permutation testing was used with 1000 permutations of the samples to establish significance, with α = 0.05. For the expression data, we log-transformed the normalized data and performed correlation analysis across all samples between each of the genes in the study. Significance was determined by assuming a normal distribution in the log-transformed expression levels and applying Student's t-distribution with an alpha of 0.05. All analyses were performed using Matlab.

## Results

### A Novel Integrative Epigenetic Approach to Screen for CTAs and Related Epigenetically Regulated Genes

**Figure 1:**
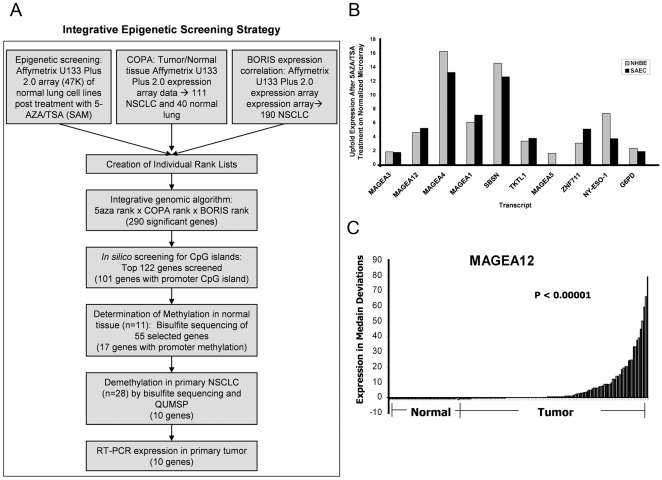
Integrative epigenetic screening strategy. (A) Schematic outline of the integrative epigenetic screening strategy utilized in this study which combined the pharmacologic demethylation of normal cell lines, comparative COPA anaylsis in primary tissue and correlation of gene expression with the epigenetic regulator BORIS in primary tissue. (B) Representative COPA graph of *MAGEA12* demonstrating the statistical approach used to find candidate overexpressed CTAs and related genes. Difference in tumor versus normal expression was significant, p value<0.00001 as measured by Mann-Whitney U test. (C) Upfold regulation of mRNA expression in treated normal lung cell lines, NHBE and SAEC, as measured by Affymetrix Human Genome U133 Plus 2.0 mRNA expression platform.

We developed an integrative, high-throughput approach to screen for CTAs and other coordinately expressed genes based on three key previously published factors: (1) CTAs are expressed in germline cells and many tumors, but not in normal somatic tissue [1], [21], (2) CTAs have promoter CpG islands that are methylated and silenced in normal somatic tissue, but, experimentally, can be expressed by promoter demethylation [1], [10], [11], [12] and (3) the transcription factor BORIS has been shown to induce de-repression of several CTAs in NSCLC and other tumor/tissue types (Figure 1A) [22], [23], [24], [25].

The first arm of our screening approach involved the pharmacologic demethylation of 2 normal lung cell lines, Normal Human Bronchial Epithelial (NHBE) and Human Small Airway Epithelial (SAEC) cells (Lonza, Walkersville, MD), using a 5-aza/TSA treatment protocol that has previously been successful in defining candidate tumor suppressor genes by demethylating tumor cell lines. With the understanding that CTAs are silenced by methylation in normal tissue, we used normal cell lines to identify genes that are typically repressed in normal tissues, but can be re-expressed by pharmacologic manipulation. Two normal lung cell lines, NHBE and SAEC, were treated with 5 µM 5-aza deoxycytidine for 72 hours and Trichostatin A for 24 hours prior to harvesting total RNA for expression array analysis using the Affymetrix Human Genome U133 Plus 2.0 expression platform. These results were then analyzed using dChip and Significance Analysis of Microarrays (SAM) [17], [18]. Genes were ranked based on their SAM score(d). SAM also reported the fold change in the mean expression of the target genes in the 5-aza/TSA treatment group versus the control group (Figure 1B).

We concurrently analyzed data from 40 normal lung and 111 NSCLC expression microarrays from expO datasets (all run on the Affymetrix Human Genome U133 Plus 2.0 mRNA expression platform) publically available online as part of the Gene Expression Omnibus (GEO/NCBI). For our analysis of these 151 primary tissue expression array data sets, we used a technique known as Cancer Outlier Profile Analysis (COPA). COPA is a method to search for marked overexpression of particular genes that occur only in a subset of cases, whereas traditional analytical methods based on standard statistical measures fail to find genes with this type of expression profile [19]. COPA was a particularly useful method for us to search for CTAs and genes with similar expression profiles based on previous studies showing that CTAs are heterogeneously expressed both across a wide patient population and within individual tumor specimens [26], [27], [28]. Genes with a normal tissue COPA expression scaled score >2 at the 95th percentile were eliminated from the rank list. All remaining genes were then ranked based on their COPA score at the 90^th^ percentile; statistical significance of the expression differences in the COPA diagrams were measured by Mann-Whitney U test (Figure 1C).

For the final arm of our screening approach, we used the previous data set with 111 NSCLC and an additional expO dataset with 79 additional NSCLC primary tumor tissues also run on the Affymetrix Human Genome U133 Plus 2.0 mRNA expression platform. In these 190 primary NSCLC samples, we correlated BORIS expression patterns within each tumor with expression of all transcripts incorporated in the U133 Plus 2.0 array by calculating a correlation coefficient using Excel. All genes were then ranked based on the strength of the correlation between their expression and that of BORIS expression across all 190 samples.

Three rank lists were produced by ranking genes by SAM score(d) following 5-aza/TSA treatment in normal lung cell lines, COPA score in primary tissue, and BORIS correlation in primary tissue. These 3 rank lists were combined by using a rank product (x*y*z). Using a significance threshold (α = 0.005) and subsequent random permutations of our rank-lists, we identified 290 genes that were significantly differentially upregulated based on epigenetic screening and tissue microarray expression patterns (Table S2) [29].

Initially, an *in silico* approach utilizing MethPrimer was used to confirm the presence of CpG islands in the promoter regions of our top candidates [20]. The top 100 of the 290 significant genes as well as 22 genes selected based on biological relevance in cancer related pathways were selected to be screened via this approach, and 101 were found to contain 1 or more promoter CpG islands.

**Figure 2:**
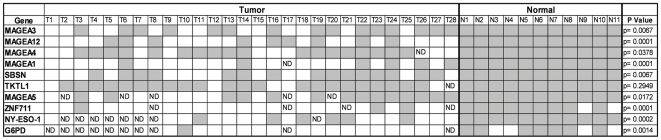
Promoter methylation status in primary tissues. Shown are the bisulfite sequencing results with associated p values in 28 NSCLC tumor samples and 11 normal lung tissues for: *MAGEA3* (13/28, p = 0.0067), *MAGEA12* (19/28, p = 0.0001), *MAGEA4* (9/27, p = 0.0378), *MAGEA1* (21/27, p = 0.0001), *SBSN* (13/28, p = 0.0067), *TKTL1* (5/27, p = 0.2949), *MAGEA5* (9/23, p = 0.0172), *ZNF711* (21/24, p = 0.0001), *NY-ESO-1* (14/20, p = 0.0002), *G6PD* (17/18, p = 0.0014). In parentheses are the ratio of demethylated promoters in tumors and the p-values as calculated by the Fisher's exact test comparing demethylation in tumors vs. normals. Shaded boxes represent methylated promoters, ND = methylation status not determined by bisulfite sequencing.

We then used a separate cohort of 11 normal lung tissues from patients without a cancer diagnosis to confirm epigenetic silencing via promoter methylation in normal lung mucosa from patients without a lung neoplasm. Bisulfite sequencing of CpG islands in the promoter regions of 55 selected gene targets with CpG islands was used to determine the methylation status. Only 17/55 promoter regions demonstrated complete methylation at all sequenced CpG sites in all or nearly all of the normal tissues (Table S2). These targets were subsequently bisulfite sequenced in a separate cohort of 28 primary NSCLC to search for the presence of promoter hypomethylation. Of these remaining targets, 10/17 showed promoter demethylation in some fraction of tumors including: *MAGEA3* (13/28, p = 0.0067), *MAGEA12* (19/28, p = 0.0001), *MAGEA4* (9/27, p = 0.0378), *MAGEA1* (21/27, p = 0.0001), *SBSN* (13/28, p = 0.0067), *TKTL1* (5/27, p = 0.2949), *MAGEA5* (9/23, p = 0.0172), *ZNF711* (21/24, p = 0.0001), *NY-ESO-1* (14/20, p = 0.0002), *G6PD* (17/18, p = 0.0014), (Fisher's exact test, two-sided) (Table 1 and Figure 2).

**Table 1:** List of 10 genes elucidated by our integrative epigenetic screening approach and found to be coordinately expressed and demethylated in NSCLC.

Symbol	Description	Integrated Rank Position	COPA Score*	Upregulated with 5-Aza Average Fold Change (SAM Score (d))	Promoter CpG Island Present	Methylated in Normal Lung Tissue	Unmethylated in NSCLC Tumor Tissue
MAGEA3	Melanoma antigen family A, 3	4	60.6	1.8 (9.9)	Y	Y	Y
MAGEA12	Melanoma antigen family A, 12	5	24.1	4.8 (7.8)	Y	Y	Y
MAGEA4	Melanoma antigen family A, 4	6	101.0	14.9 (11.3)	Y	Y	Y
MAGEA1	Melanoma antigen family A, 1	11	12.8	6.5 (10.5)	Y	Y	Y
SBSN	Suprabasin	27	6.4	13.5 (31.3)	Y	Y	Y
TKTL1	Transketolase-like 1	35	1.8	3.5 (15.3)	Y	Y	Y
MAGEA5	Melanoma antigen family A, 5	41	16.9	1.5 (3)	Y	Y	Y
ZNF711	Zinc finger protein 6	43	10.0	3.8 (9.3)	Y	Y	Y
NY-ESO-1	Cancer/testis antigen 1B	72	117.6	5.9 (4)	Y	Y	Y
G6PD	Glucose-6-phosphate dehydrogenase	105	22.4	2.1 (10.6)	Y	Y	Y

* Tumor COPA score at 90^th^ percentile.

Transcriptional upregulation of target genes after 5-aza/TSA treatment in our cell line system was confirmed using quantitative RT-PCR on the 5-aza/TSA-treated normal cells compared to mock-treated cells for these 10 genes (Figure S1). Each gene with the exception *MAGEA12* demonstrated significant upregulation by 5-aza/TSA treatment in at least one cell line supporting functional gene regulation by promoter hypomethylation.

### CTAs and Associated Genes Are Coordinately Demethylated and Expression Is Correlated with Promoter Demethylation

**Figure 3:**
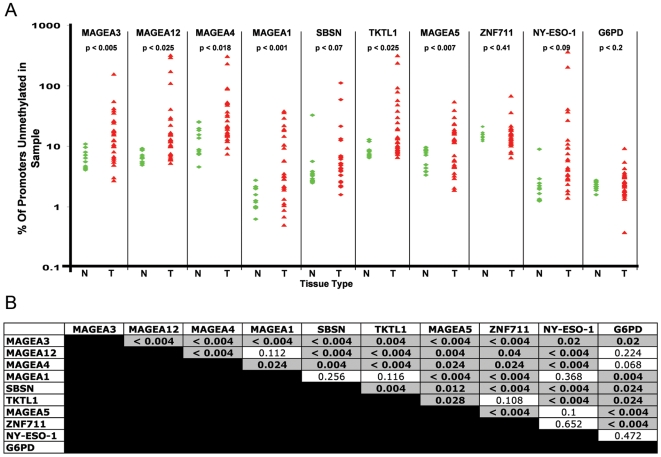
CTAs and associated genes are coordinately demethylated. (A) QUMSP was conducted in our cohort of 28 NSCLC and 11 normal lung tissues. Experiments were performed in triplicate, mean values are shown representing the percentage of unmethylated promoters on a logarithmic scale. Statistically significant tumor-specific demethylation was found in *MAGEA3*, *MAGEA12* (p<0.025), *MAGEA4* (p<0.018), *MAGEA1* (p<0.001), *TKTL1* (p<0.025) and *MAGEA5* (p<0.007). Two additional targets slightly missed significance at α<0.05, *SBSN* (p<0.07) and *NY-ESO-1* (p<0.09) (2 tailed Student's t-test assuming unequal variances). (B) Promoter hypomethylation (QUMSP) correlation p-value matrix for NSCLC (n = 28; Spearman's correlation permutation test). Shaded cells represent significant p-values.

In order to confirm the bisulfite sequencing results in our target genes and to provide a dataset of continuous variables to express the status of promoter demethylation, we devised a rapid, quantitative assay for specifically measuring non-methylated promoters, which we termed Quantitative Unmethylation-Specific PCR (QUMSP). We assayed DNA extracted from our cohort of 28 primary NSCLC tumor samples and 11 normal lung samples from non-cancer patients (Figure 3A). Significant tumor-specific demethylation was found in *MAGEA3* (p<0.005), *MAGEA12* (p<0.025), *MAGEA4* (p<0.018), *MAGEA1* (p<0.001), *TKTL1* (p<0.025) and *MAGEA5* (p<0.007). Two additional targets slightly missed significance at α<0.05, *SBSN* (p<0.07) and *NY-ESO-1* (p<0.09) (2 tailed Student's t-test assuming unequal variance).

Given the tumor-specific demethylation pattern seen for our target genes, we next wanted to determine if demethylation of the promoter regions of these genes occurred in a coordinated fashion within tumor samples. We utilized the Spearman's correlation permutation testing to determine significant coordinated demethylation using the QUMSP results from our cohort of 28 NSCLC (Figure 3B). The p-value matrix of the Spearman's correlation coefficient shows that for any of the target genes, demethylation tended to coordinately occur with a minimum of 6 of the other genes. Shaded cells represent significant p-values. This offers evidence that demethylation is highly associated with coordinated regulation of these CTAs and related target genes in NSCLC, and strongly suggests an epigenetic mechanism of activation.

In order to confirm tumor specific expression of our target genes, we used quantitative RT-PCR to determine mRNA expression in our cohort of NSCLC and normal lung tissue (Figure S2A−J). Six genes had significantly increased expression in tumors *MAGEA12* (p<0.02), *SBSN* (p<0.002), *TKTL1* (p<0.02), *ZNF711* (p<0.008), *NY-ESO-1* (p<0.001), *G6PD* (p<0.006). Three genes slightly missed significance at the α<0.05 level: *MAGEA3* (p<0.09), *MAGEA4* (p<0.06) and *MAGEA1* (p<0.08) (2 tailed Student's t-test assuming unequal variance).

We next wanted to determine if demethylation was responsible for the derepression of the CTAs and related genes in NSCLC. Four target genes showed a significant positive correlation between mRNA expression (quantitative RT-PCR) and promoter hypomethylation (QUMSP): *MAGEA12* (p = 0.024), *MAGEA4* (p<0.004), *SBSN* (p = 0.004) and *NY-ESO-1* (p<0.004) (Figure S3A−D) (Spearman's correlation permutation test). *TKTL1* (p = 0.1), *MAGEA5* (p = 0.104) and *MAGEA3* (p = 0.2) also showed a positive correlation between demethylation and expression, but missed significance (Table S3). These data suggest demethylation of promoter regions is partially responsible for the regulation of the majority of our target genes.

### CTAs and Associated Target Genes Are Coordinately Expressed

**Figure 4:**
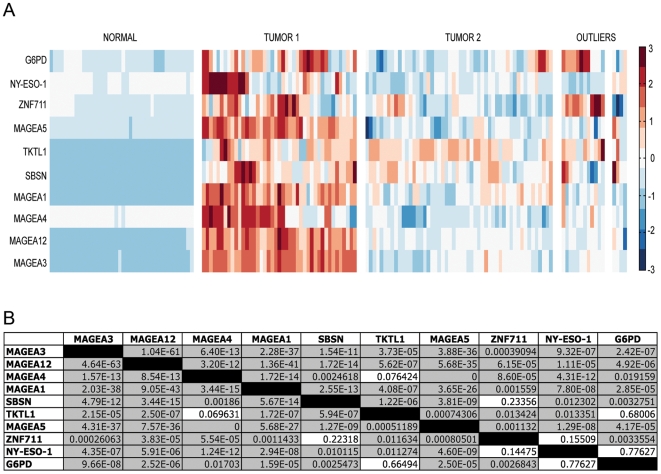
CTAs and associated target genes are coordinately expressed in NSCLC. (A) Heat map of transcript expression as measured by the Affymetrix Human Genome U133 Plus 2.0 mRNA expression platform. The data were first normalized by setting the mean and standard deviation of each gene to 0 and 1 respectively across all 151 samples. The data were then clustered in the sample domain using hierarchical clustering with average linkage and a Euclidean distance metric. (B) Pearson's correlation coefficient p-value matrix for gene expression which tests for the coexpression of each gene pair across all tumors. This comparison shows the expression correlation of each gene pair in 111 NSCLC. Values to the upper right have been corrected with the Benjamin Hochberg multiple test correction to decrease the false discovery rate; uncorrected values are displayed in the lower left. Shaded cells represent significant p-values.

Given the findings in the previous analyses of our cohort of primary tissue showing that these target genes are differentially expressed in tumors, their promoter regions are coordinately demethylated within tumors and expression is correlated with demethylation, we examined our initial cohort of 111 tumors assayed using the Affymetrix Human Genome U133 Plus 2.0 mRNA expression platform to determine if our target genes were coordinately expressed within tumor samples in this large sample set. Figure 4A shows a heat map of transcript expression as measured by the U133 Plus 2.0 array for 40 normal lung samples from non-cancer patients and 111 NSCLC primary tissue samples. This figure visualizes the relationship of expression between the 10 target genes in normal and tumor samples. The data were first normalized by setting the mean and standard deviation of each gene to 0 and 1 respectively across all 151 samples. The data were then clustered in the sample domain using hierarchical clustering with average linkage and a Euclidean distance metric. This provided three clusters: 1) all 40 normal samples, 2) 43 of the 111 tumor samples showing high expression in most of the genes and strong correlations between them (Tumor 1), and 3) 52 of 111 tumor samples showing lower expression but with expression still above normals (Tumor 2). The remaining 16 of 111 tumor samples did not cluster together or in these groups. This analysis not only provided confirmation that expression of our target genes is limited to a subset of tumors with little or no expression in the normal tissue, but also, that these targets appear to be coordinately expressed in a large subset, 38.7%, of these tumors.

In addition, the data show that there is a distinct correlation between the coordinated expression of these 10 target genes and the squamous cell carcinoma (SCC) subtype of NSCLC. In the group Tumor 1 72% of the samples were of the SCC subtype, whereas only 23% of tumors in Tumor group 2 were SCCs, p<0.00001 (Fisher's exact test, two-sided), OR = 8.4, 95% CI 3.1−24.3.

To formally test the coordinate expression of these genes, we next constructed a p-value matrix derived from the Pearson's correlation coefficients calculated between the expression levels of each target (Figure 4B). Values to the upper right have been corrected with the Benjamin Hochberg multiple test correction to decrease the false discovery rate; however, there is no change in significance after this correction (uncorrected values displayed in lower left). Shaded cells represent significant p-values. Using this pairwise comparison method, we found highly significant coordinated upregulation of all 10 target genes within a subset of tumor samples.

### *SBSN* and *NY-ESO-1* Are Growth Promoting in Normal and NSCLC Cell Lines

**Figure 5:**
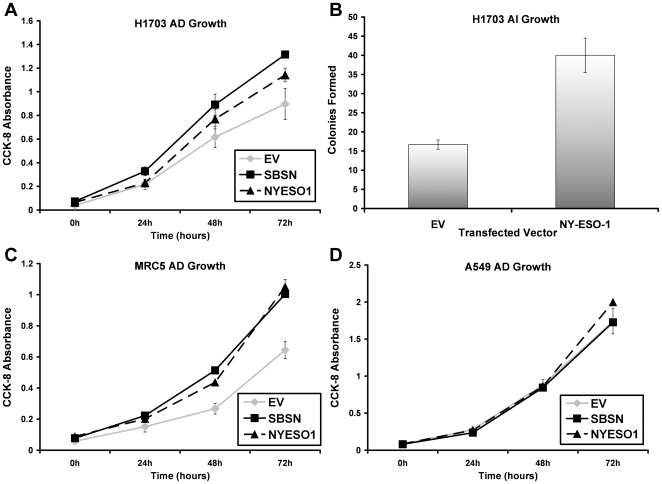
*SBSN* and *NY-ESO-1* are growth promoting in normal and NSCLC cell lines. (A) Anchorage dependent growth following transient transfection of *NY-ESO-1* or *SBSN* construct into NCI-H1703 cells (at 72 hours, 23% (±12%) and 47% (±6.5%) growth increase, respectively). (B) Anchorage independent growth was assayed in NCI-H1703 cells following transfection with empty vector (EV) or *NY-ESO-1*. *NY-ESO-1* significantly increased the number of colonies formed, 40 versus 16 (p<0.03) (2 tailed Student's t-test assuming unequal variance). (C) Anchorage dependent growth following transient transfection of *NY-ESO-1* or *SBSN* construct into normal lung fibroblast cells,MRC-5, (at 72 hours, 63% (±14.5%) and 56% (±5%) growth increase, respectively). (D) Anchorage dependent growth following transient transfection of *NY-ESO-1* or *SBSN* construct into lung adenocarinoma cells, A549, (neither gene caused a significant increase in growth at 72 hours).

We performed transient transfections in multiple NSCLC and normal cell lines in order to gain insight into the function of *NY-ESO-1* and newly identified *SBSN*, two genes that showed significant correlation between promoter hypomethylation and transcriptional upregulation in primary tumor tissue. In lung squamous cell carcinoma cell lines NCI-H1703 and NCI-H226, transient transfection of a construct containing either *NY-ESO-1* or *SBSN* caused a significant increase in anchorage dependent (AD) cell growth. In H1703 cells at 72 hours, *NY-ESO-1* and *SBSN* caused a 23% (±12%) and 47% (±6.5%) growth increase, respectively (Figure 5A). In H226 cells at 72 hours, *NY-ESO-1* and *SBSN* caused a 24% (±12%) and 42% (±4%) growth increase, respectively (Figure S4A). In addition, both target genes were able to induce increased AD growth in a normal lung fibroblast cell line, MRC-5. At 72 hours post transfection, *NY-ESO-1* induced a 63% (±14.5%) increase in growth while *SBSN* induced a 56% (±5%) increase in growth (Figure 5C). Interestingly, neither gene caused an increase in AD growth in lung adenocarcinoma cell line A549 (Figure 5D).

In order to further evaluate the growth promoting potential of these target genes, anchorage independent (AI) growth in soft agar was measured following transient transfection with either *SBSN* or *NY-ESO-1* in NCI-H1703 and MRC-5 cell lines. Because the target gene constructs used contained a G418 resistance cassette, all AI growth assays were performed in the presence of G418. After 12 days in soft agar, H1703 cells transfected with *NY-ESO-1* formed significantly more colonies than their empty vector transfected counterparts averaging 40 colonies versus 16 colonies, respectively (p<0.03) (2 tailed Student's t-test assuming unequal variance) (Figure 5B). Although *SBSN* was able to cause a small increase in colony formation, 19 versus 16, this data did not reach significance (Figure S4B). Neither *SBSN nor NY-ESO-1* were able to transform the normal lung fibroblast cell line, MRC-5 (data not shown).

## Discussion

The development of vaccines aimed at inducing an active specific cytotoxic immune response in NSCLC has been met with many challenges, including the lack of suitable target antigens [16]. Although NY-ESO-1 and MAGEA3 are currently undergoing clinical trials in various human malignancies, including NSCLC, the use of single antigen vaccine formulations have often been met with limited clinical outcomes [14], [15], [16], [30]. The identification of multiple, tumor specific antigens may facilitate the use of more effective polyvalent cancer vaccines; however, this requires the identification of multiple aberrantly expressed genes, as well as an understanding of the patterns of expression that are prevalent in the tumor of interest [15], [16], [31].

In this study, we used a novel, integrative epigenetic screening approach to specifically look for coordinately expressed genes in human NSCLC whose transcription is driven by promoter demethylation. From the over 47,000 transcripts incorporated in the Affymetrix Human Genome U133 Plus 2.0 expression platform, we were able to identify 10 genes that showed both differential overexpression and promoter region hypomethylation in NSCLC. Surprisingly, 6 of the 10 genes discovered via this approach were known CT-X antigens, *MAGEA3*, *MAGEA12, MAGEA4, MAGEA1*, *MAGEA5* and *NY-ESO-*1. Four additional CTAs, *MAGEA9, MAGEA6, MAGEB2 and CT45-2*, were within the top 20 on our rank list; however, these genes did not meet our screening selection criteria due to failure to show complete methylation of promoter regions in our separate cohort of normal lung tissue by bisulfite sequencing (Table S2). It is possible that, by using less stringent selection criteria, we would identify these and other genes as differentially expressed, albeit with incomplete promoter methylation patterns in normal lung tissue. In this study we selected only the top 55 of the 290 possible targets identified after integrative analysis in a single solid tumor type for further analysis. It is our expectation that further investigation of the remaining genes, as well as the use of normal cell lines and tumors derived from additional tissue types in an integrative approach, will allow for discovery of additional, novel, epigenetically-controlled genes that may also show coordinated expression in tumors and serve as possible targets for screening and immunogenic therapy.

Although some of the CTAs we identified using this technique have previously been shown to be expressed to some degree in NSCLC; the demonstration of a high degree of coordinated expression in a large sample set related to epigenetic unmasking is a novel finding [26], [31], [32], [33], [34], [35], [36]. In a previous study of 19 lung carcinoma cell lines expressing various MAGEA family members, there was nearly complete concordance between the RT-PCR and IHC results [34]. Thus the use of quantitative RT-PCR is a valid method for detecting CT antigen expression, especially when dealing with primary tissue where it is usually not possible to isolate sufficient quantities of protein for analysis. In addition, this same study showed that 44% of the 187 NSCLC samples tested on tissue microarrays stained positive, to some degree, for MAGEA family expression, supporting the fact that CTAs are expressed at the protein level in NSCLC [34].

Four target genes showed a significant positive correlation between mRNA expression and promoter hypomethylation, *MAGEA12, MAGEA4*, *SBSN* and *NY-ESO-1* (Figure S3A−D). *TKTL1*, *MAGEA5* and *MAGEA3* also showed a positive correlation between demethylation and expression, but missed significance (Table S3). The lack of correlation between expression and hypomethylation in some of our target genes is expected given the fact that multiple other mechanisms such as point mutations, insertions, deletions and loss of heterozygosity could be involved in gene expression regulation in NSCLC. Alternatively, a larger sample size may facilitate the ability to define a closer association between promoter methylation status and expression in these genes.

In addition to the 6 mentioned CT-X antigens, our discovery approach elucidated 4 additional target genes that we showed to be coordinately expressed with the known CTAs and demethylated in tumors. Interestingly, three of these 4 genes are encoded by the X chromosome, *TKTL-1, ZNF-711* and *G6PD*. *TKTL1* has been correlated with worse outcomes in patients with invasive colon and urothelial tumors, oxygen-independent glucose usage and validated as a potential biomarker in breast cancer [37], [38]. *SBSN, ZNF-711* and *G6PD* have not previously been associated with tumor specific expression or carcinogenesis.

We were also able to define a subset of NSCLC that coordinately and aberrantly expressed these target genes. The tight association of this subset with a squamous histology supports the concept that tumors that express this gene cluster have a distinct phenotype, and may preferentially express this cohort of cancer testes antigens due to selection pressures.

CTAs are attractive targets for tumor immunotherapy because of their restricted expression patterns in normal human tissue. Currently, demethylating agents and HDAC inhibitors are being studied as adjuvant treatment options for NSCLC and other human malignancies, and combinations of these drugs continue to undergo bench-top and clinical investigation [39], [40], [41]. These epigenetic therapies are being utilized based on data that suggests that methylation of tumor suppressor genes plays a fundamental role in tumor formation, progression, and recurrence after resection. Promoter region methylation of certain genes in resected NSCLC specimens was recently shown to be associated with recurrence of the tumor and poorer patient outcomes [42]. An additional study has previously shown that *NY-ESO-1* and *MAGEA3* are upregulated in a proportion of patients treated with 5-aza-2′-deoxycytidine in cancers involving the lung, esophagus, or pleura [41]. With our data suggesting that multiple CTAs are coordinately expressed in NSCLC and demethylation coordinately upregulates multiple known CTAs and associated genes from our target list, it might be useful in the future to combine the use of demethylating agents with immunotherapy targeted against these genes that might be derepressed after treatment with 5-AZA and other demethylating agents. Targeting multiple CTAs that are coordinately expressed would help to improve the efficacy seen with monovalent immunologic agents.

To date, the function of these genes expressed uniquely in NSCLC has not been well explored. There are data that indicate that MAGEA family members have growth promoting effects, and CTA members have been associated with biologic pathways that support a malignant phenotype. In this study, we showed that both *NY-ESO-*1 and newly identified *SBSN* are growth promoting in lung squamous cell carcinoma cell lines and normal lung fibroblasts. Interestingly, neither gene product induced increased cell growth in the lung adenocarcinoma cell line, supporting the concept that these genes are tightly associated with the squamous histologic subtype and their expression causes a selective growth advantage in these cells. In addition we were able to show that *NY-ESO-1* was able to induce a significant increase in anchorage independent cell growth, and, while, *SBSN* increased soft agar growth, it was not significant (Figure 5 and Figure. S4). In the future, we believe that additional analyses of other genes that are aberrantly expressed via promoter demethylation in NSCLC would be expected to demonstrate functional effects that contribute to carcinogenesis, and may identify other potential candidate proto-oncogenes that are activated via promoter demethylation. Recent data in head and neck squamous cell carcinoma indicates that similar strategies are effective in identifying candidate proto-oncogenes [43].

Using an integrative analysis combining pharmacologic demethylation and previously published primary tissue array data, we have defined a common epigenetic mechanism for the coordinated expression of CTAs and defined additional targets that may serve as targets for immunotherapy. In the future, the combined use of coordinately expressed CTAs and related genes could aid in the design of more effective, polyvalent immunotherapeutic protocols in NSCLC and other tumor types, as well as identification of potential therapeutic targets and candidate proto-oncogenes in NSCLC.

## Supporting Information

Figure S1 Promoter demethylation causes transcriptional upregulation. Relative fold upregulation after treatment with 5-aza/TSA is shown in NHBE and SAEC cell lines as measured by quantitative RT-PCR. The ratio of 5-aza/TSA treated expression to baseline with associated p values are shown for MAGEA3, MAGEA4, MAGEA12, MAGEA1, SBSN, TKTL1, MAGEA5, ZNF711, NY-ESO-1 and G6PD. Experiments were performed in triplicate, the values are means ± s.d. Each gene, with the exception MAGEA12, demonstrated significant upregulation by 5-aza/TSA treatment in at least one cell line (2 tailed Student's t-test assuming unequal variance).
(2.14 MB TIF)

Figure S2 Target gene expression is upregulated in NSCLC vs. normal lung tissues. (A–J) Quantitative RT-PCR in a cohort of 28 NSCLC and 5 normal lung tissues. Significant increased expression in tumors was seen for MAGEA12 (p<0.02), SBSN (p<0.002), TKTL1 (p<0.02), ZNF711 (p<0.008), NY-ESO-1 (p<0.001), G6PD (p<0.006). Three genes slightly missed significance at the α<0.05 level: MAGEA3 (p<0.09), MAGEA4 (p<0.06) and MAGEA1 (p<0.08) (2 tailed Student's t-test assuming unequal variance). Experiments were performed in triplicate, values are mean ± s.d.
(2.26 MB PPT)

Figure S3 Gene transcript expression is correlated with promoter demethylation. (a–d) Scatter plots showing Log2 QRT-PCR values plotted against Log2 QUMSP for 28 NSCLC and 5 normal lung tissues. Significant positive correlation between mRNA expression and promoter hypomethylation were seen for MAGEA12 (p = 0.024), MAGEA4 (p<0.004), SBSN (p = 0.004) and NY-ESO-1 (p<0.004) (Spearman's correlation permutation test).
(1.72 MB TIF)

Figure S4 SBSN and NY-ESO-1 induce AD and AI growth in lung SCC cell lines. (A) Anchorage dependent growth following transient transfection of NY-ESO-1 or SBSN construct into NCI-H226 cells (at 72 hours, 24% (±12%) and 42% (±4%) growth increase, respectively). (B) Anchorage independent growth was assayed in NCI-H1703 cells after transfection with empty vector (EV), SBSN, or NY-ESO-1. Both SBSN and NY-ESO-1 induced an increase in the number of colonies formed, but only NY-ESO-1 reached significance (p<0.03) (2 tailed Student's t-test assuming unequal variance).
(0.34 MB TIF)

Table S1 Primer Sequences
(0.02 MB XLS)

Table S2 Supplementary Table 2. List of the 290 significant genes found after combing the three rank ordered lists (- = Not determined).
(0.06 MB PDF)

Table S3 Spearman's correlation permutation test p value table showing the strength of positive correlation between demethylation (QUMSP) and mRNA expression (RT-PCR).
(0.34 MB TIF)
