# Supplementary figures and images for: Integrative Discovery of Epigenetically Derepressed Cancer Testis Antigens in NSCLC
Source: PLoS One. 2009 Dec 4;4(12):e8189. doi: 10.1371/journal.pone.0008189 (PMC2781168; doi:10.1371/journal.pone.0008189)

## Slide 1
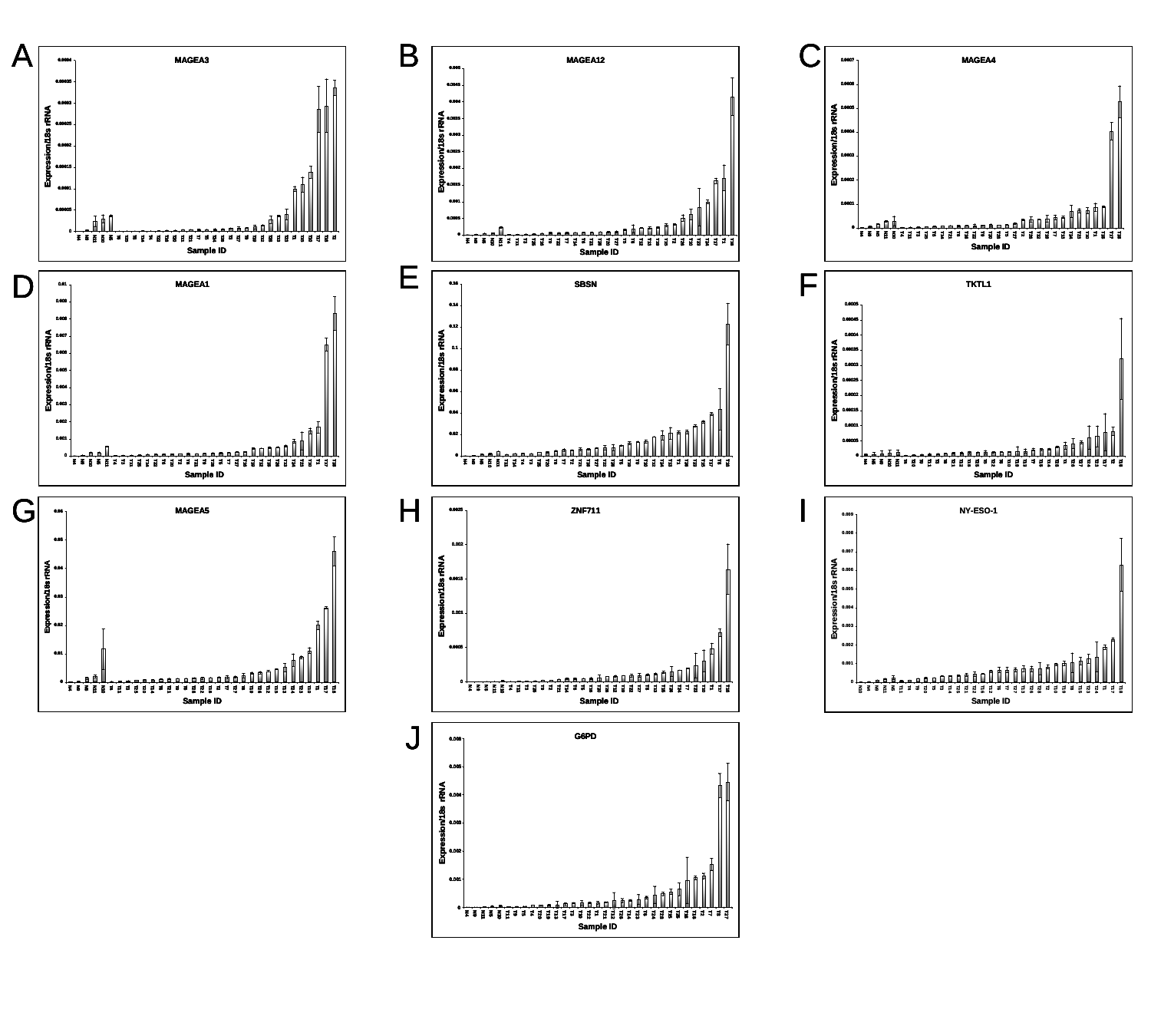

Supplement: Figure S2 — Target gene expression is upregulated in NSCLC vs. normal lung tissues. (A–J) Quantitative RT-PCR in a cohort of 28 NSCLC and 5 normal lung tissues. Significant increased expression in tumors was seen for MAGEA12 (p<0.02), SBSN (p<0.002), TKTL1 (p<0.02), ZNF711 (p<0.008), NY-ESO-1 (p<0.001), G6PD (p<0.006). Three genes slightly missed significance at the α<0.05 level: MAGEA3 (p<0.09), MAGEA4 (p<0.06) and MAGEA1 (p<0.08) (2 tailed Student's t-test assuming unequal variance). Experiments were performed in triplicate, values are mean ± s.d. (2.26 MB PPT) [file pone.0008189.s002.ppt]
