# Supplementary material for: Integrative Discovery of Epigenetically Derepressed Cancer Testis Antigens in NSCLC
Source: PLoS One. 2009 Dec 4;4(12):e8189. doi: 10.1371/journal.pone.0008189 (PMC2781168; doi:10.1371/journal.pone.0008189)
Supplement: Table S2 — Supplementary Table 2. List of the 290 significant genes found after combing the three rank ordered lists (- = Not determined). (0.06 MB PDF) [file pone.0008189.s006.pdf]

Combined Rank	Gene Name	Accession	Promoter CpG Island	Methylated in Normals	Demethylated in Tumors
1	fibronectin type III and SPRY dor	NM_024333	yes	no	
2	melanoma antigen family A, 9	BC002351	yes	yes	no
3	hypothetical protein FLJ14627	AW299700	yes	no	
4	melanoma antigen family A, 3	BC000340	yes	yes	yes
5	melanoma antigen family A, 12	BC003408	yes	yes	yes
6	melanoma antigen family A, 4	AW438674	yes	yes	yes
7	melanoma antigen family A, 6	U10691	yes	yes	no
8	HRAS-like suppressor	NM_020386	yes	no	
9	HRAS-like suppressor	NM_020386	yes	no	
10	kinesin family member 1A	AL533416	no		
11	melanoma antigen family A, 1 (di	NM_004988	yes	yes	yes
12	Zic family member 2 (odd-paired	AF193855	yes	no	
13	hypothetical LOC388727	AI382195	yes		
14	T-cell lymphoma invasion and me	U90902	yes	no	
15	Interleukin 17 receptor D	AW007080	yes	yes	no
16	cancer/testis antigen CT45-2 /// c	U31738	yes	yes	no
17	chromosome 7 open reading fran	AI884867	yes	no	
18	keratin 23 (histone deacetylase ir	NM_015515	no		
19	melanoma antigen family B, 2	NM_002364	yes		
20	small proline-rich protein 1A	AI923984	no		
21	FXYD domain containing ion tran	NM_022006	yes		
22	hypothetical protein FLJ38736	Z98443	yes	no	
23	keratin 6B	AI831452	no		
24	troponin T1, skeletal, slow	AJ011712	yes		
25	oxoglutarate dehydrogenase-like	NM_018245	yes	no	
26	UDP glycosyltransferase 8 (UDP	N22272	yes	no	
27	suprabasin	AI814274	yes	yes	yes
28	hypothetical protein FLJ10781	NM_018215	yes	no	
29	ribonuclease, RNase A family, 7	AJ131212	no		
30	tubulin, beta polypeptide paralog	AL533838	yes	no	
31	Clone IMAGE:110436 mRNA sec	AW444944	no		
32	zinc finger DAZ interacting protei	BG502305	yes	no	
33	Ras association (RalGDS/AF-6) c	AW070877	yes		
34	Adaptor-related protein complex	AA205444	no		
35	transketolase-like 1	Z49258	yes	yes	yes
36	G antigen 4 /// G antigen 5 /// G a	NM_001476	yes	yes	no
37	synaptogyrin 3	NM_004209	yes	no	
38	hypothetical protein MGC23280	AI076793	yes	no	
39	reticulon 3	BE544689	yes	no	
40	cytochrome P450, family 24, sub	NM_000782	yes	no	
41	melanoma antigen family A, 5	AI200443	yes	yes	yes
42	protocadherin 19	AB037734	yes	no	
43	zinc finger protein 6 (ZNF711)	AU157017	yes	yes	yes
44	small proline-rich protein 2A	NM_006945	no		
45	LOC441335	AI343600	yes		
46	fibroblast growth factor 19	AF110400	yes	no	
47	potassium voltage-gated channe	NM_000238	yes		
48	small proline-rich protein 3	BF575466	no		
49	integrin beta 1 binding protein (m	NM_012278	yes	yes	no

50	similar to hypothetical protein A2	AW188087	no		
51	calbindin 1, 28kDa	NM_004929	yes		
52	galactokinase 1	BG474736	yes		
53	CDNA FLJ35846 fis, clone TEST	AK093165	yes		
54	Ring finger protein 32	BE962709	yes	no	
55	Hypothetical protein FLJ13744	NM_025011	yes		
56	dimethylarginine dimethylaminoh	NM_013974	yes		
57	G antigen 2 /// G antigen 4 /// G a	NM_001474	yes		
58	MARCKS-like 1	NM_023009	yes		
59	small nuclear RNA activating com	NM_003084	yes	no	
60	UDP glycosyltransferase 8 (UDP	NM_003360	yes		
61	gb:BF446127 /DB_XREF=gi:115	BF446127	yes		
62	peripherin	NM_006262	yes		
63	retinol binding protein 1, cellular	NM_002899	yes		
64	DKFZP564I1171 protein	BF528646	yes		
65	cytidine deaminase	NM_001785	yes		
66	RAB3B, member RAS oncogene	BC005035	yes		
67	protein phosphatase 1, regulatory	AK024593	yes	no	
68	fatty acid binding protein 7, brain	NM_001446	no		
69	dimethylarginine dimethylaminoh	AJ012008	yes		
70	Zinc finger and BTB domain cont	BC003066	yes		
71	keratin 17	NM_000422	no		
72	cancer/testis antigen 1B (NY-ES(	AF038567	yes	yes	yes
73	opposite strand transcription unit	AL831967	yes		
74	hypothetical protein FLJ14503	AW237462	no		
75	keratin 6B	L42612	no		
76	Homo sapiens, clone IMAGE:522	BC037977	no		
77	matrix metalloproteinase 13 (colla	NM_002427	no		
78	ovary-specific acidic protein	AF329088	yes	no	
79	hypothetical protein MGC50844	AK000208	yes		
80	myosin VB	AI991160	yes		
81	keratin 14 (epidermolysis bullosa	BC002690	yes		
82	adducin 2 (beta)	NM_017488	yes		
83	Homo sapiens, clone IMAGE:529	AW665239	yes		
84	hypothetical protein FLJ20489	NM_017842	yes		
85	coagulation factor C homolog, co	AA669336	yes		
86	SET and MYND domain containi	NM_022743	no		
87	proenkephalin	NM_006211	yes		
88	enolase 2 (gamma, neuronal)	NM_001975	yes		
89	neuronal cell adhesion molecule	NM_005010	yes	no	
90	homeo box A10	AI375919	yes		
91	neurofilament, heavy polypeptide	X15306	yes		
92	amine oxidase (flavin containing)	BE348688	yes		
93	lactotransferrin	NM_002343	yes		
94	neurotensin	NM_006183	no		
95	calbindin 1, 28kDa	AW014927	yes		
96	gb:AI692575 /DB_XREF=gi:4969	AI692575	no		
97	interleukin 21 receptor	AF269133	yes		
98	keratin 13	NM_002274	no		
99	galanin receptor 2	AF080586	yes	no	
100	GRB2-associated binding protein	NM_012296	yes		
101	peptidyl arginine deiminase, type	NM_016233	-		

102	chromosome 6 open reading fran	NM_030568	-		
103	synaptotagmin XI	AA626780	yes		
104	matrix metalloproteinase 10 (stro	NM_002425	yes	yes	no
105	glucose-6-phosphate dehydroger	NM_000402	yes	yes	yes
106	neurofilament, heavy polypeptide	NM_021076	-		
107	zinc finger protein 38	AL136865	-		
108	gamma-glutamyltransferase-like	AL049709	-		
109	GDP-mannose 4,6-dehydratase	NM_001500	-		
110	Homo sapiens, clone IMAGE:483	AI862542	-		
111	glycine C-acetyltransferase (2-an	NM_014291	-		
112	adenosine deaminase	X02189	-		
113	similar to hepatocellular carcinom	AI625022	yes	no	
114	brevican	AI739071	-		
115	NADH dehydrogenase (ubiquinor	BF434335	-		
116	SRY (sex determining region Y)-I	NM_000346	yes	no	
117	Hypothetical protein FLJ35801	BG260069	-		
118	Kell blood group precursor (McLe	NM_021083	-		
119	keratin associated protein 2-1 ///	BC012486	-		
120	actin like protein	BF594459	-		
121	pleckstrin homology domain cont	AI056683	-		
122	NSE1 /// hypothetical LOC40094	AI601101	-		
123	calmegin	NM_004362	-		
124	hypothetical LOC151760	BC038577	-		
125	potassium voltage-gated channel	AB044806	-		
126	small proline-rich protein 1B (cor	NM_003125	no		
127	UL16 binding protein 2	AA831769	-		
128	hypothetical protein MGC4825	AF061264	-		
129	Transcribed locus, weakly similar	AA629068	-		
130	quiescin Q6	NM_002826	yes		
131	hypothetical protein LOC196394	BG031897	-		
132	protein phosphatase 2 (formerly 2	AI669212	-		
133	prostaglandin-endoperoxide synt	S36219	-		
134	CDNA clone IMAGE:4620359, pa	BC023568	-		
135	hypothetical LOC344595	AA463827	-		
136	hypothetical protein FLJ32942	BG150433	-		
137	Ras association (RalGDS/AF-6) (	AI167789	yes	no	
138	Hypothetical protein FLJ10385	U58658	-		
139	ephrin-A3	AW189015	yes	no	
140	homeo box A1	S79910	-		
141	melanoma antigen family F, 1	NM_022149	yes	no	
142	hydroxysteroid (17-beta) dehydro	NM_002153	-		
143	lipidosin	BE856376	-		
144	hypothetical protein FLJ23322	NM_024955	-		
145	gb:BF241692 /DB_XREF=gi:111	BF241692	-		
146	hypothetical protein LOC112868	BF447901	-		
147	protein phosphatase 1, regulatory	NM_006741	-		
148	B-box and SPRY domain contain	AJ276691	-		
149	endothelin 1	BC036851	yes	no	
150	hypothetical protein LOC285590	BC038561	-		
151	distal-less homeobox 4	NM_001934	-		
152	transcription factor Dp-2 (E2F dir	NM_006286	-		
153	gb:NM_022443.1 /DB_XREF=gi:	NM_022443	-		

154	dispatched homolog 2 (Drosophil	AB051529	-		
155	gb:BF511718 /DB_XREF=gi:115	BF511718	-		
156	WNK lysine deficient protein kina	H06509	-		
157	FERM, RhoGEF (ARHGEF) and	BF725250	-		
158	LOC441297	AI870903	-		
159	KIAA1967	BC003172	-		
160	neurofilament, light polypeptide 6	AL537457	-		
161	immunoglobulin superfamily, mer	AB037776	-		
162	family with sequence similarity 50	NM_012135	-		
163	protein phosphatase 1J (PP2C d	AW117553	-		
164	dimethylarginine dimethylaminoh	AK026191	-		
165	brain-specific angiogenesis inhibi	NM_001703	-		
166	hairy and enhancer of split 2 (Dro	AK023754	-		
167	Homeo box D13	AI971104	-		
168	prostaglandin-endoperoxide synt	BE613133	-		
169	adenosine deaminase	NM_000022	-		
170	membrane protein, palmitoylated	NM_005374	-		
171	quinolinate phosphoribosyltransfe	NM_014298	-		
172	cholinergic receptor, nicotinic, alp	BC000513	-		
173	Hypothetical protein FLJ14627	AL120375	-		
174	Insulin-like growth factor binding	AL522781	-		
175	RAB3B, member RAS oncogene	NM_002867	yes	no	
176	keratin 8 /// keratin 8	U76549	-		
177	keratin 17	Z19574	-		
178	acid phosphatase-like 2	AW069729	-		
179	glutamate decarboxylase 1 (brain	NM_000817	-		
180	transmembrane protease, serine	NM_014058	-		
181	hypothetical protein LOC112868	AI935915	-		
182	cytochrome P450, family 4, subfa	NM_000896	-		
183	histone deacetylase 5	NM_005474	yes	no	
184	antigen p97 (melanoma associate	BC001875	-		
185	RNA-binding region (RNP1, RRM	AL109955	-		
186	cadherin 2, type 1, N-cadherin (n	M34064	-		
187	cyclin-dependent kinase 5	NM_004935	yes	no	
188	v-myb myeloblastosis viral oncog	NM_005375	yes	no	
189	Zinc finger protein 192	AI920953	-		
190	Transmembrane, prostate androg	AI821781	-		
191	Wiskott-Aldrich syndrome-like	BF438330	yes	no	
192	Full-length cDNA clone CS0DK0	AI521166	-		
193	ubiquitin carboxyl-terminal estera	NM_004181	-		
194	dopa decarboxylase (aromatic L-	NM_000790	-		
195	ankyrin repeat and SOCS box-co	AI872284	-		
196	Full length insert cDNA clone YR	AI919519	-		
197	Doublecortin and CaM kinase-like	AI129626	-		
198	nicotinamide nucleotide adenylylt	AF288395	-		
199	growth differentiation factor 11	AF028333	-		
200	lipid phosphate phosphatase-rela	NM_022737	-		
201	kinesin light chain 2-like	AI279514	-		
202	ATPase, Ca++ transporting, card	AI220427	-		
203	SH3-domain binding protein 4	AF015043	-		
204	Neurofilament, light polypeptide 6	BF055311	-		
205	zinc finger protein 42	AF450454	-		

206	alcohol dehydrogenase 7 (class I	U07821	-		
207	glycine amidinotransferase (L-arg	X86401	-		
208	normal mucosa of esophagus spe	AF228422	-		
209	testis expressed sequence 15	AL133653	yes	no	
210	Fraser syndrome 1	AL157471	-		
211	cancer/testis antigen 1B	AJ275978	yes		
212	hypothetical gene supported by E	AI569804	-		
213	NAD(P)H dehydrogenase, quinon	NM_000904	yes		
214	Full-length cDNA clone CS0DF01	BG260087	-		
215	DNA segment on chromosome 4	NM_014392	-		
216	hypothetical protein FLJ23033	NM_024686	-		
217	Phosphatidylinositol-specific phos	AW170015	-		
218	small proline-rich protein 1A	NM_005987	-		
219	gb:AW274658 /DB_XREF=gi:666	AW274658	-		
220	tumor suppressor candidate 3	AI884858	-		
221	signal transducer and activator of	NM_007315	-		
222	chromosome X open reading fran	NM_017863	-		
223	B-box and SPRY domain contain	NM_017688	-		
224	triple functional domain (PTPRF i	BF223718	-		
225	acheron	BF792126	-		
226	tubulin, beta 4	AL567012	-		
227	guanylate cyclase activator 1A (r	L36861	-		
228	hypothetical protein FLJ22595	NM_025047	-		
229	cornifelin /// cornifelin	AB049591	-		
230	glutathione peroxidase 2 (gastroi	NM_002083	-		
231	glutaminase 2 (liver, mitochondria	NM_013267	-		
232	SLAM family member 9	NM_033438	-		
233	KIAA1211 protein	BE855799	-		
234	Retinol binding protein 1, cellular	AI802099	-		
235	hypothetical protein FLJ38736	AW299463	-		
236	nerve growth factor receptor (TN	NM_002507	-		
237	Similar to expressed sequence A	AI971535	-		
238	hypothetical protein LOC256021	AK055439	-		
239	chromogranin B (secretogranin 1	NM_001819	-		
240	serine (or cysteine) proteinase in	NM_002575	-		
241	hypothetical protein FLJ30707	AW450586	-		
242	translocase of outer mitochondria	NM_014820	yes	no	
243	DKFZP434B0335 protein	BE616972	-		
244	SWI/SNF related, matrix associat	AI760760	-		
245	dehydrogenase/reductase (SDR	AL547782	-		
246	glucosidase, beta, acid 3 (cytoso	NM_020973	-		
247	serine protease inhibitor, Kazal ty	NM_003122	-		
248	Ribosomal protein L4	BF431260	-		
249	prostaglandin-endoperoxide synt	NM_000962	-		
250	Myeloid leukemia factor 1	AI911434	-		
251	hypothetical protein LOC254128	BF939830	-		
252	hypothetical protein LOC283901	AK095480	-		
253	protein phosphatase 1H (PP2C d	AB032983	-		
254	carnitine palmitoyltransferase 1C	AL565745	-		
255	cytochrome P450, family 39, sub	AI796334	-		
256	carboxypeptidase E	AI922855	-		
257	glutathione peroxidase 7	AA406605	-		

258	ovostatin 2	AW594320	yes		
259	WD repeat domain 9	AI638279	-		
260	phospholipase D1, phophatidylch	U38545	-		
261	suppression of tumorigenicity 13	BE866412	-		
262	phorbol-12-myristate-13-acetate-	NM_021127	-		
263	piggyBac transposable element c	NM_024554	-		
264	Kringle containing transmembran	BF221745	-		
265	tripartite motif-containing 37	AK022701	-		
266	calmodulin-like 5	NM_017422	-		
267	chromosome 9 open reading fran	BC031861	-		
268	nuclear factor (erythroid-derived	NM_004289	-		
269	gb:AI436290 /DB_XREF=gi:4309	AI436290	-		
270	myo-inositol 1-phosphate synthas	AL137749	-		
271	PTEN induced putative kinase 1	BF432478	-		
272	gb:BE615699 /DB_XREF=gi:989	BE615699	-		
273	proopiomelanocortin (adrenocorti	NM_000939	-		
274	testes-specific protease 50	NM_013270	yes	no	
275	IGF-II mRNA-binding protein 3	NM_006547	-		
276	homeo box D10	AW299531	-		
277	elongation of very long chain fatty	AF292387	-		
278	KIAA1718 protein	AK026372	-		
279	prostaglandin-endoperoxide synt	NM_000962	-		
280	cortexin 1	BF982289	-		
281	progestagen-associated endome	NM_002571	-		
282	ubiquitin specific protease 2	AW274034	-		
283	Transcription factor CP2-like 3	AI224578	-		
284	gb:AL049569 /DB_XREF=gi:526	AL049569	-		
285	cytoplasmic linker 2 /// cytoplasm	BC006259	-		
286	gb:AI743489 /DB_XREF=gi:5111	AI743489	-		
287	hypothetical locus LOC283861	BC009880	-		
288	G protein-coupled receptor 160	BC000181	-		
289	inorganic pyrophosphatase 2	BF446912	-		
290	glutamate decarboxylase 1 (brain	NM_013445	-		
